# Supplementary material for: A Likelihood Approach for Real-Time Calibration of Stochastic Compartmental Epidemic Models
Source: PLoS Comput Biol. 2017 Jan 17;13(1):e1005257. doi: 10.1371/journal.pcbi.1005257 (PMC5240920; doi:10.1371/journal.pcbi.1005257)
Supplement: S1 File — (TAR.GZ) [file pcbi.1005257.s014.tar.gz › HSPH_Online-SI-Revision/EnKF07/n10-extreme/n10-extreme_EnKF2_table-8.pdf]

1	newly 3 weeks spec	116.	953.02	{72., 2146.}	738.707	0.915897	newly 3 weeks cumul	174.	4730.15	{1337., 8868.}	2625.56	1.37466	newly 5 weeks spec	547.	463.65	{0., 1004.}	61.1499	0.705045	newly 5 weeks cumul	988.	5854.95	{2198., 10 743.}	501.963	0.802067	newly 66 weeks cumul	9069.	7169.44	{3015., 11 513.}	38.7192	0.310013
2	newly 3 weeks spec	211.	146.54	{17., 165.}	90.4076	0.587855	newly 3 weeks cumul	450.	2440.65	{904., 6459.}	448.922	0.588273	newly 5 weeks spec	408.	34.7	{0., 33.}	96.8873	2.13055	newly 5 weeks cumul	1168.	2541.14	{921., 6473.}	138.81	0.291481	newly 62 weeks cumul	7262.	2589.32	{929., 6473.}	70.9945	0.666224
3	newly 3 weeks spec	63.	464.48	{16., 1121.}	661.492	0.787386	newly 3 weeks cumul	84.	3913.56	{471., 9145.}	4563.21	1.42037	newly 5 weeks spec	540.	301.81	{0., 1028.}	89.713	1.56038	newly 5 weeks cumul	830.	4572.19	{583., 9149.}	466.558	0.660048	newly 68 weeks cumul	9108.	5624.31	{628., 9683.}	41.3113	0.427789
4	newly 3 weeks spec	319.	98.31	{15., 161.}	77.6082	0.73935	newly 3 weeks cumul	641.	2095.76	{931., 6027.}	231.56	0.42043	newly 5 weeks spec	626.	16.31	{0., 28.}	97.3946	2.41505	newly 5 weeks cumul	1754.	2149.6	{946., 6034.}	73.6146	0.272978	newly 62 weeks cumul	8332.	2164.06	{948., 6034.}	74.0271	0.716986
5	newly 3 weeks spec	240.	744.6	{24., 1715.}	249.6	0.604839	newly 3 weeks cumul	440.	5893.02	{1900., 10 044.}	1246.55	1.06929	newly 5 weeks spec	689.	202.43	{0., 576.}	77.479	1.27959	newly 5 weeks cumul	1573.	6474.61	{2100., 11 467.}	320.65	0.606956	newly 65 weeks cumul	9428.	6760.05	{2595., 12 434.}	42.3705	0.316219
6	newly 3 weeks spec	128.	892.99	{34., 2103.}	621.977	0.910744	newly 3 weeks cumul	239.	4854.8	{1332., 9867.}	1943.05	1.25583	newly 5 weeks spec	489.	438.24	{1., 1050.}	64.5562	0.724144	newly 5 weeks cumul	1036.	5913.65	{1808., 11 129.}	484.381	0.79067	newly 66 weeks cumul	9287.	7113.32	{2892., 11 394.}	38.8472	0.338897
7	newly 3 weeks spec	131.	948.22	{32., 1935.}	650.076	0.822096	newly 3 weeks cumul	207.	4756.22	{1234., 9043.}	2198.95	1.26368	newly 5 weeks spec	469.	507.46	{0., 1156.}	80.2516	0.950631	newly 5 weeks cumul	924.	5964.35	{1518., 9663.}	548.047	0.746282	newly 67 weeks cumul	8335.	7142.01	{1532., 10 592.}	31.8264	0.210693
8	newly 3 weeks spec	187.	624.33	{107., 1576.}	253.064	0.455332	newly 3 weeks cumul	315.	4148.39	{1188., 9569.}	1218.	0.959043	newly 5 weeks spec	589.	159.05	{8., 363.}	75.3973	0.917522	newly 5 weeks cumul	1255.	4611.5	{1246., 10 741.}	274.977	0.46373	newly 64 weeks cumul	9223.	4839.11	{1310., 11 251.}	57.3385	0.466212
9	newly 3 weeks spec	245.	621.91	{100., 1636.}	182.551	0.409415	newly 3 weeks cumul	451.	4138.12	{1195., 9614.}	820.745	0.813742	newly 5 weeks spec	556.	159.89	{7., 380.}	74.25	0.935945	newly 5 weeks cumul	1389.	4602.45	{1256., 10 848.}	240.945	0.431705	newly 64 weeks cumul	8874.	4832.26	{1278., 11 329.}	57.3363	0.458625
10	newly 3 weeks spec	219.	582.9	{101., 1544.}	189.817	0.401316	newly 3 weeks cumul	414.	4083.93	{1130., 9628.}	889.234	0.847165	newly 5 weeks spec	476.	147.48	{9., 354.}	73.7731	0.87021	newly 5 weeks cumul	1223.	4512.69	{1222., 10 842.}	276.681	0.47073	newly 64 weeks cumul	8927.	4726.98	{1258., 11 341.}	57.7856	0.462907
11	newly 3 weeks spec	260.	195.92	{5., 860.}	123.362	1.0263	newly 3 weeks cumul	477.	1250.62	{182., 6452.}	234.34	0.409968	newly 5 weeks spec	539.	38.56	{0., 52.}	95.5399	2.15605	newly 5 weeks cumul	1360.	1380.01	{213., 6966.}	131.776	0.683718	newly 59 weeks cumul	8770.	1417.13	{231., 7002.}	85.5474	1.25419
12	newly 3 weeks spec	147.	606.45	{100., 1519.}	327.531	0.538072	newly 3 weeks cumul	271.	3836.94	{1092., 9266.}	1320.69	1.01661	newly 5 weeks spec	376.	165.38	{9., 355.}	65.7766	0.725119	newly 5 weeks cumul	836.	4306.74	{1242., 10 503.}	423.689	0.630762	newly 64 weeks cumul	8374.	4556.46	{1341., 10 746.}	57.3993	0.45949
13	newly 3 weeks spec	356.	324.79	{3., 1099.}	139.531	1.20577	newly 3 weeks cumul	666.	879.78	{43., 2540.}	137.721	0.637956	newly 5 weeks spec	681.	150.01	{0., 760.}	89.1233	2.11396	newly 5 weeks cumul	1797.	1269.82	{51., 4794.}	106.893	0.897712	newly 60 weeks cumul	8214.	1523.76	{65., 7897.}	81.9613	1.3591
14	newly 3 weeks spec	267.	731.41	{25., 1612.}	218.528	0.5996	newly 3 weeks cumul	466.	5513.85	{1663., 9999.}	1091.47	1.00534	newly 5 weeks spec	683.	226.32	{0., 637.}	77.1332	1.32992	newly 5 weeks cumul	1583.	6140.12	{1666., 11 457.}	298.582	0.581243	newly 65 weeks cumul	8671.	6479.53	{1666., 12 349.}	44.0112	0.325962
15	newly 3 weeks spec	284.	152.74	{15., 181.}	85.	0.658219	newly 3 weeks cumul	552.	2297.17	{904., 6454.}	323.857	0.499181	newly 5 weeks spec	600.	35.73	{0., 42.}	97.0217	2.24598	newly 5 weeks cumul	1572.	2402.46	{919., 6464.}	96.855	0.292309	newly 62 weeks cumul	7388.	2457.11	{937., 6464.}	72.2218	0.682919
16	newly 3 weeks spec	140.	700.9	{27., 1706.}	436.386	0.693991	newly 3 weeks cumul	241.	5565.69	{1640., 9922.}	2214.86	1.26496	newly 5 weeks spec	517.	189.72	{0., 552.}	78.0542	1.2825	newly 5 weeks cumul	1029.	6111.29	{1679., 11 386.}	503.824	0.739125	newly 65 weeks cumul	8875.	6377.78	{1679., 11 755.}	44.1458	0.334319
17	newly 3 weeks spec	117.	876.93	{33., 1778.}	676.829	0.810458	newly 3 weeks cumul	203.	4741.92	{1079., 9511.}	2237.16	1.24425	newly 5 weeks spec	499.	454.82	{0., 1051.}	74.7896	1.04983	newly 5 weeks cumul	983.	5833.83	{1317., 9611.}	499.95	0.715162	newly 67 weeks cumul	8786.	6925.31	{1515., 10 463.}	31.4556	0.234715
18	newly 3 weeks spec	167.	731.16	{22., 1691.}	377.521	0.709092	newly 3 weeks cumul	309.	5568.52	{1661., 10 754.}	1709.24	1.17376	newly 5 weeks spec	657.	219.38	{0., 574.}	76.9893	1.33443	newly 5 weeks cumul	1340.	6181.1	{1662., 11 515.}	371.904	0.653649	newly 65 weeks cumul	9346.	6505.05	{1662., 12 420.}	44.1589	0.347534
19	newly 3 weeks spec	61.	950.95	{40., 1940.}	1473.89	1.04612	newly 3 weeks cumul	87.	4763.74	{1240., 9030.}	5375.66	1.63308	newly 5 weeks spec	390.	507.34	{0., 1149.}	97.9077	0.977295	newly 5 weeks cumul	643.	5980.66	{1522., 9664.}	832.137	0.899359	newly 67 weeks cumul	8626.	7163.63	{1535., 10 588.}	30.7847	0.206183
20	newly 3 weeks spec	181.	606.96	{100., 1528.}	254.475	0.481964	newly 3 weeks cumul	336.	3838.21	{1082., 9302.}	1048.37	0.935022	newly 5 weeks spec	555.	166.58	{8., 364.}	74.0577	0.848383	newly 5 weeks cumul	1224.	4310.48	{1216., 10 542.}	262.528	0.488116	newly 64 weeks cumul	9145.	4562.25	{1305., 10 766.}	58.1996	0.481658
21	newly 3 weeks spec	233.	31.62	{0., 71.}	86.7983	1.48222	newly 3 weeks cumul	468.	505.08	{145., 976.}	61.6795	0.295703	newly 5 weeks spec	546.	7.99	{0., 17.}	98.5366	2.62304	newly 5 weeks cumul	1440.	527.68	{158., 977.}	65.4722	0.574936	newly 61 weeks cumul	8562.	538.83	{159., 977.}	93.7067	1.33205
22	newly 3 weeks spec	42.	456.85	{19., 1098.}	1005.55	0.857933	newly 3 weeks cumul	65.	3839.18	{458., 9153.}	5810.03	1.50825	newly 5 weeks spec	339.	296.39	{0., 1023.}	115.956	1.48697	newly 5 weeks cumul	532.	4488.14	{561., 9174.}	753.632	0.786041	newly 68 weeks cumul	9331.	5539.21	{595., 9773.}	42.958	0.449277
23	newly 3 weeks spec	289.	282.69	{0., 743.}	94.9792	1.09179	newly 3 weeks cumul	567.	3696.14	{742., 8722.}	556.543	0.685544	newly 5 weeks spec	502.	83.92	{0., 281.}	87.2749	1.98801	newly 5 weeks cumul	1436.	3923.96	{767., 10 167.}	190.844	0.409932	newly 63 weeks cumul	6906.	4103.94	{767., 10 167.}	60.7576	0.453128
24	newly 3 weeks spec	396.	42.35	{0., 65.}	95.6035	2.03628	newly 3 weeks cumul	794.	664.22	{132., 1365.}	64.2292	0.379719	newly 5 weeks spec	721.	26.01	{0., 11.}	98.3897	3.01252	newly 5 weeks cumul	2068.	721.41	{146., 1435.}	73.2693	0.675514	newly 61 weeks cumul	8926.	809.57	{149., 1463.}	91.2192	1.27363
25	newly 3 weeks spec	77.	638.19	{114., 1566.}	732.325	0.778303	newly 3 weeks cumul	116.	4043.27	{1189., 9342.}	3386.32	1.40175	newly 5 weeks spec	441.	177.04	{12., 354.}	65.8458	0.715931	newly 5 weeks cumul	781.	4545.03	{1326., 10 525.}	486.716	0.671091	newly 64 weeks cumul	9000.	4812.09	{1384., 10 743.}	55.397	0.428833
26	newly 3 weeks spec	130.	896.24	{34., 1811.}	619.538	0.800228	newly 3 weeks cumul	214.	4554.66	{1115., 8740.}	2031.15	1.21222	newly 5 weeks spec	577.	480.55	{0., 1182.}	65.6308	1.01754	newly 5 weeks cumul	1076.	5704.08	{1319., 9591.}	438.913	0.688538	newly 67 weeks cumul	7335.	6812.93	{1426., 10 526.}	35.6918	0.245477
27	newly 3 weeks spec	133.	727.96	{27., 1725.}	480.391	0.706451	newly 3 weeks cumul	225.	5791.54	{1648., 10 087.}	2476.75	1.31469	newly 5 weeks spec	482.	204.42	{0., 637.}	79.61	1.25439	newly 5 weeks cumul	995.	6374.68	{1707., 11 502.}	546.874	0.754196	newly 65 weeks cumul	9083.	6700.28	{1866., 12 425.}	42.1949	0.290514
28	newly 3 weeks spec	69.	493.16	{6., 1235.}	645.478	0.807007	newly 3 weeks cumul	105.	3602.57	{463., 8951.}	3335.32	1.29201	newly 5 weeks spec	530.	332.62	{0., 1013.}	92.0038	1.54692	newly 5 weeks cumul	861.	4331.66	{571., 8972.}	421.659	0.631853	newly 68 weeks cumul	9312.	5496.59	{587., 9785.}	43.6569	0.452014
29	newly 3 weeks spec	156.	285.7	{0., 762.}	177.667	1.07686	newly 3 weeks cumul	228.	3493.94	{709., 8937.}	1433.96	1.00309	newly 5 weeks spec	612.	86.22	{0., 288.}	86.9706	2.08772	newly 5 weeks cumul	1151.	3731.67	{737., 10 670.}	244.126	0.44883	newly 63 weeks cumul	9014.	3873.88	{740., 11 743.}	66.8726	0.593579
30	newly 3 weeks spec	209.	336.19	{0., 764.}	146.99	1.02568	newly 3 weeks cumul	375.	3915.17	{735., 10 032.}	947.405	0.864346	newly 5 weeks spec	521.	102.71	{0., 307.}	85.7678	1.85268	newly 5 weeks cumul	1271.	4193.87	{766., 10 769.}	246.784	0.465332	newly 63 weeks cumul	8143.	4402.36	{766., 11 760.}	61.3089	0.496697
31	newly 3 weeks spec	173.	942.81	{34., 2102.}	468.272	0.782143	newly 3 weeks cumul	303.	4735.58	{678., 9697.}	1474.11	1.118	newly 5 weeks spec	607.	480.58	{6., 1022.}	56.86	0.659596	newly 5 weeks cumul	1297.	5884.9	{1459., 11 163.}	367.331	0.676627	newly 66 weeks cumul	9186.	7356.31	{2859., 12 460.}	37.3378	0.296652
32	newly 3 weeks spec	258.	134.81	{25., 224.}	84.7636	0.631985	newly 3 weeks cumul	565.	691.07	{254., 1264.}	80.4018	0.269675	newly 5 weeks spec	577.	52.45	{3., 116.}	90.9099	1.48437	newly 5 weeks cumul	1557.	825.76	{309., 1502.}	79.7148	0.557145	newly 58 weeks cumul	8222.	928.3	{327., 1557.}	88.7096	1.15429
33	newly 3 weeks spec	126.	925.4	{63., 2096.}	651.635	0.829482	newly 3 weeks cumul	184.	4897.58	{1285., 9631.}	2566.9	1.34853	newly 5 weeks spec	733.	443.88	{0., 1011.}	57.9209	0.810286	newly 5 weeks cumul	1177.	5976.51	{1497., 10 571.}	415.498	0.711224	newly 66 weeks cumul	9187.	7221.29	{3069., 11 313.}	37.2834	0.284844
34	newly 3 weeks spec	135.	499.76	{8., 1534.}	315.778	0.694217	newly 3 weeks cumul	190.	3525.72	{449., 8893.}	1761.09	1.02974	newly 5 weeks spec	568.	318.58	{0., 1004.}	84.2923	1.51963	newly 5 weeks cumul	1068.	4241.76	{557., 8909.}	323.378	0.574284	newly 68 weeks cumul	8375.	5441.89	{584., 9772.}	42.4909	0.440153
35	newly 3 weeks spec	297.	318.07	{0., 795.}	109.411	1.19782	newly 3 weeks cumul	580.	3872.55	{747., 10 052.}	572.884	0.692094	newly 5 weeks spec	563.	97.19	{0., 300.}	87.3694	2.03908	newly 5 weeks cumul	1559.	4136.16	{764., 10 772.}	186.421	0.412635	newly 63 weeks cumul	8157.	4325.53	{764., 11 761.}	61.625	0.499905
36	newly 3 weeks spec	233.	27.9	{0., 64.}	88.7725	1.62673	newly 3 weeks cumul	424.	571.5	{148., 1121.}	83.3208	0.322752	newly 5 weeks spec	562.	5.72	{0., 9.}	98.9822	2.90961	newly 5 weeks cumul	1395.	590.65	{157., 1260.}	66.7233	0.566701	newly 61 weeks cumul	9215.	599.95	{165., 1260.}	93.4894	1.35682
37	newly 3 weeks spec	197.	617.72	{104., 1561.}	233.949	0.455014	newly 3 weeks cumul	298.	3895.44	{1141., 9318.}	1212.21	0.983373	newly 5 weeks spec	605.	167.99	{11., 359.}	75.638	0.864671	newly 5 weeks cumul	1277.	4373.31	{1281., 10 537.}	252.831	0.473873	newly 64 weeks cumul	9224.	4631.09	{1348., 10 778.}	58.4498	0.477577
38	newly 3 weeks spec	40.	913.97	{44., 1977.}	2194.83	1.15667	newly 3 weeks cumul	62.	4825.64	{1130., 9550.}	7683.29	1.77807	newly 5 weeks spec	491.	478.85	{0., 1161.}	78.0591	0.975856	newly 5 weeks cumul	699.	5978.05	{1409., 9724.}	757.298	0.856749	newly 67 weeks cumul	8849.	7057.06	{1530., 10 778.}	32.1514	0.223933
39	newly 3 weeks spec	103.	913.97	{39., 2001.}	809.932	0.865327	newly 3 weeks cumul	146.	4829.88	{1145., 9495.}	3208.84	1.41071	newly 5 weeks spec	645.	477.83	{0., 1156.}	65.4713	1.03876	newly 5 weeks cumul	1060.	5980.21	{1412., 9705.}	466.912	0.683443	newly 67 weeks cumul	9368.	7055.29	{1557., 10 759.}	31.4365	0.227731
40	newly 3 weeks spec	276.	669.73	{95., 2045.}	174.185	0.418481	newly 3 weeks cumul	519.	4195.74	{1058., 9310.}	715.087	0.780104	newly 5 weeks spec	547.	169.88	{7., 410.}	72.7751	0.877264	newly 5 weeks cumul	1464.	4692.37	{1099., 10 533.}	234.196	0.455136	newly 64 weeks cumul	8627.	4941.26	{1248., 10 750.}	55.3407	0.446449
41	newly 3 weeks spec	249.	280.92	{0., 789.}	119.711	1.33889	newly 3 weeks cumul	416.	3748.27	{722., 9512.}	806.267	0.80352	newly 5 weeks spec	680.	83.45	{0., 212.}	90.4162	2.26834	newly 5 weeks cumul	1527.	3974.16	{739., 11 836.}	184.464	0.416733	newly 63 weeks cumul	9091.	4152.33	{754., 11 851.}	63.6954	0.563003
42	newly 3 weeks spec	386.	112.79	{20., 151.}	84.1062	0.911304	newly 3 weeks cumul	794.	657.61	{250., 707.}	82.0542	0.500833	newly 5 weeks spec	673.	42.06	{1., 77.}	93.7504	1.67411	newly 5 weeks cumul	2037.	767.21	{303., 895.}	83.2405	0.784257	newly 58 weeks cumul	8720.	858.65	{320., 1088.}	90.5065	1.31717
43	newly 3 weeks spec	32.	464.48	{16., 1121.}	1368.75	0.992234	newly 3 weeks cumul	43.	3913.56	{471., 9145.}	9003.81	1.69373	newly 5 weeks spec	315.	301.81	{0., 1028.}	119.165	1.44751	newly 5 weeks cumul	461.	4572.19	{583., 9149.}	900.887	0.857774	newly 68 weeks cumul	9060.	5624.31	{628., 9683.}	41.2123	0.426412
44	newly 3 weeks spec	255.	281.05	{4., 835.}	141.949	0.999046	newly 3 weeks cumul	527.	1801.48	{178., 7598.}	311.389	0.518609	newly 5 weeks spec	597.	57.06	{0., 94.}	92.6968	2.11289	newly 5 weeks cumul	1543.	1976.34	{209., 7811.}	145.375	0.70784	newly 59 weeks cumul	8693.	2061.26	{215., 7818.}	80.297	1.1443
45	newly 3 weeks spec	293.	305.89	{0., 749.}	101.382	1.05758	newly 3 weeks cumul	562.	3823.74	{736., 8940.}	584.964	0.699326	newly 5 weeks spec	606.	89.37	{0., 281.}	89.0776	2.0665	newly 5 weeks cumul	1576.	4069.01	{776., 10 231.}	177.46	0.392581	newly 63 weeks cumul	8765.	4237.27	{776., 11 090.}	63.5705	0.524933
46	newly 3 weeks spec	273.	23.72	{0., 62.}	91.3114	1.83018	newly 3 weeks cumul	518.	604.93	{169., 1123.}	66.1139	0.284489	newly 5 weeks spec	572.	5.65	{0., 11.}	99.0122	2.83766	newly 5 weeks cumul	1495.	621.77	{176., 1314.}	66.1973	0.547364	newly 61 weeks cumul	8858.	630.92	{180., 1332.}	92.8774	1.29028
47	newly 3 weeks spec	140.	278.97	{0., 769.}	189.764	1.04638	newly 3 weeks cumul	227.	3464.06	{717., 8952.}	1427.59	0.999777	newly 5 weeks spec	505.	84.11	{0., 290.}	87.1584	1.99447	newly 5 weeks cumul	1027.	3694.45	{743., 10 675.}	275.909	0.466401	newly 63 weeks cumul	9302.	3833.82	{749., 11 735.}	67.3455	0.609163
48	newly 3 weeks spec	115.	954.65	{77., 2111.}	746.635	0.923316	newly 3 weeks cumul	204.	4728.9	{1397., 8753.}	2225.4	1.3106	newly 5 weeks spec	483.	465.98	{2., 1039.}	65.1884	0.652884	newly 5 weeks cumul	959.	5859.4	{2165., 10 801.}	520.448	0.81436	newly 66 weeks cumul	9126.	7178.5	{3000., 11 528.}	38.5354	0.30992
49	newly 3 weeks spec	85.	913.97	{44., 1977.}	994.459	0.915538	newly 3 weeks cumul	133.	4825.64	{1130., 9550.}	3528.93	1.4499	newly 5 weeks spec	380.	478.85	{0., 1161.}	99.6553	0.971542	newly 5 weeks cumul	694.	5978.05	{1409., 9724.}	763.445	0.859742	newly 67 weeks cumul	8859.	7057.06	{1530., 10 778.}	32.1355	0.224022
50	newly 3 weeks spec	88.	640.93	{23., 1303.}	654.716	0.75294	newly 3 weeks cumul	156.	5676.27	{1661., 9971.}	3542.12	1.45846	newly 5 weeks spec	376.	166.53	{0., 405.}	80.4601	1.25862	newly 5 weeks cumul	733.	6160.82	{1663., 10 527.}	747.138	0.870628	newly 65 weeks cumul	9078.	6363.87	{1663., 11 693.}	42.3427	0.315465
